# Reverse Micellar Dyeing of Cotton Fabric with Reactive Dye Using Biodegradable Non-Ionic Surfactant as Nanoscale Carrier: An Optimisation Study by One-Factor-at-One-Time Approach

**DOI:** 10.3390/polym15204175

**Published:** 2023-10-20

**Authors:** Yiu Lun Alan Tang, Shixin Jin, Cheng Hao Lee, Ho Shing Law, Jiali Yu, Yanming Wang, Chi-wai Kan

**Affiliations:** 1 School of Fashion and Textiles, The Hong Kong Polytechnic University, Hung Hom, Kowloon, Hong Kong; yluntang@polyu.edu.hk (Y.L.A.T.); chenghao.lee@polyu.edu.hk (C.H.L.); shing-john.law@connect.polyu.hk (H.S.L.); jiali.yu@polyu.edu.hk (J.Y.); amyym.wang@polyu.edu.hk (Y.W.); 2 Dental Material Science, Division of Applied Oral Sciences and Community Dental Care, Faculty of Dentistry, The University of Hong Kong, Pokfulam, Hong Kong; jasonjin@connect.hku.hk; 3 Research Centre for Resources Engineering towards Carbon Neutrality, The Hong Kong Polytechnic University, Hung Hom, Kowloon, Hong Kong

**Keywords:** Tergitol surfactant, secondary alcohol ethoxylate, cotton fabric, reactive dyes, non-aqueous dyeing, reverse micelle, octane, salt-free

## Abstract

This study investigates the feasibility of using biodegradable secondary alcohol ethoxylate (SAE) non-ionic surfactant as a building block for the formation of reverse micelles, functioning as reactive dye carriers for the dyeing of cotton fabric in non-aqueous octane medium. Ten dyeing parameters were optimised, by a one-factor-at-a-time approach, namely: (i) effect of colour fixation agent; (ii) surfactant-to-water mole ratio; (iii) surfactant-to-co-surfactant mole ratio; (iv) volume of soda ash; (v) volume of dye; (vi) solvent-to-cotton ratio; (vii) dyeing temperature; (viii) dyeing time; (ix) fixation time; (x) soda-ash-to-cotton ratio. The colour properties, fastness properties and physical properties of SAE-dyed samples were experimentally compared with the conventional water-dyed samples. The optimised condition was found when SAE samples were dyed as follows: (a) 1:20 surfactant-to-water ratio; (b) 1:8 surfactant-to-co-surfactant ratio; (c) 10:1 solvent ratio; (d) 40 min dyeing time; (e) 60 min fixation time; and (f) 70 °C dyeing and fixation temperature. The results showed that SAE-dyed samples have better colour strength, lower reflectance percentage and comparable levelness, fastness and physical properties than that of water-dyed samples. SEM images revealed that the dyed cotton fibres had no severe surface damage caused by an SAE-based reverse micellar dyeing system. The TEM image depicts that the reverse micelle was of nanoscale, spherical-shaped and had a core–shell structure, validating the presence of reverse micelle as a reactive dye carrier and the potential of an SAE-based reverse micellar system for dyeing of cotton fabrics.

## 1. Introduction

Textile dyeing is one of the most important processes in the textile industry to produce a value-added aesthetic appeal of textile products for human consumption. The conventional textile dyeing process, however, poses an undesirable threat to the environment, in which it consumes a million tons of water while it generates substantial effluent discharges containing residual dyes, chemicals, salts and alkalis with high pH, BOD and COD values [1,2,3].

To address the environmental concerns from the public, different sustainable and novel dyeing approaches for cotton fabric have been found in the literature. Periyasamy [4] and Jabar et al. [5] used bio-mordant and natural dye from syzygium cumini fruit extracts and mangiferin for sustainable dyeing of cotton fabric, respectively. Supercritical carbon dioxide has been applied as a medium for dyeing of cotton fabric with newly synthesised dye [6,7]. Pei et al. [8] reported the use of a silicon emulsion system for non-aqueous salt-free dyeing of cotton. Wei et al. [9] investigated the use of hydrophobic deep eutectic solvent (HDES) composed of thymol and menthol for dyeing of cotton. Attempts have also been made to chemically modify cotton substrates for salt-free dyeing with anionic dyes [10,11]. In addition, Mamun Kabir et al. [12] examined the use of dioctyl sodium sulfosuccinate surfactant for low-liquor-ratio dyeing of cotton.

Apart from those methods, the use of surfactant as a building block for reverse micelle formation as a reactive dye carrier in non-aqueous solvent medium is also one of the promising ways to achieve a salt-free and water-saving approach for dyeing of cotton fabric [13]. Surfactant, also known as surface active agent, is an amphiphilic molecule comprising two distinct structural moieties in which one is polar (hydrophilic) while another is nonpolar (hydrophobic). Owing to its amphiphilic property, it may either be incorporated as micelle in aqueous phase or self-assembled as reverse micelles in nonpolar oil phase once when the surfactant concentration is above the critical micelle concentration (CMC) [14,15].

Reverse micelles are nano-spherical aggregates self-assembled by surfactants in non-aqueous medium with the ability to solubilise a small amount of water, forming a water pool in their interior region [16]. They were first applied in cotton dyeing with reactive dye by the use of anionic surfactant, Aerosol-OT, as their building block [17]. However, the uneven aqueous microenvironment caused by the ionic functional groups of anionic surfactant shifts the selection of surfactant towards non-ionic in nature. Yi et al. [18] and Yi et al. [19] then reported the use of non-ionic surfactant, Triton X-100, for reverse micelle formation. Nevertheless, due to the presence of the aromatic group and poor biodegradability of Triton X-100, it has been regarded as an environmentally unfriendly surfactant which is forbidden, especially in European countries [20,21].

Our previous works focused on the use of non-ionic polyethylene glycol (PEG)-based [22] and alkyl polyglucoside (APG)-based [23] surfactants and rhamnolipid biosurfactant [24] for reverse micellar dyeing of cotton fabric in a different non-aqueous solvent medium. Compared with poly(ethylene glycol) (12) tridecyl ether (PEG-12), which is a mixture of C_11_ to C_14_ iso-alkyl ethers, Tergitol type 15-S-12 (T15S12) is a secondary alcohol ethoxylate (SAE) with distinctive branched hydrophobic tails [25], and it is readily biodegradable and specially created to meet the “designed to degrade” principle [21,26]. To the best of our knowledge, using T15S12 SAE-based biodegradable non-ionic surfactant (HLB value of 14.5) as a dye carrier for reverse micellar dyeing of cotton fabric is unknown and has not yet been explored and found in the literature.

In this study, the feasibility of using T15S12 non-ionic surfactant as a building block for reverse micelle formation and as a reactive dye carrier for dyeing of cotton fabric is investigated. Several purposes of this work include: (a) to optimise the parameters for reverse micellar dyeing of cotton fabric; (b) to compare the colour properties of the SAE-dyed fabrics with water-dyed fabrics in terms of colour yield, levelness, reflectance and CIE L*a*b* values; (c) to examine the surface damage of the SAE-dyed fabric; (d) to observe the morphology of dye-encapsulated reverse micelle formed by T15S12 surfactant; and (e) to assess the colourfastness properties of SAE-dyed fabrics.

## 2. Materials and Methods

### 2.1. Materials and Reagents

**Figure 1:**
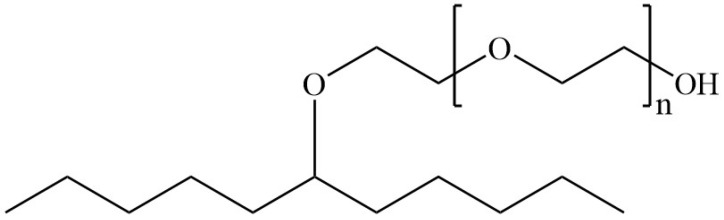
The chemical structure of Tergitol type 15-S-12 secondary alcohol ethoxylated (SAE) biodegradable surfactant (n = 12) (HLB value of 14.5).

Commercially available ready-for-dyeing 100% cotton woven fabric (density: 127 ends and picks per cm; weight: 139 g/m^2^) was pre-washed with 2 g/L of home laundry detergent at 49 °C for 45 min, tumble-dried and conditioned for 24 h at standard environment (20 ± 2 °C and 65 ± 2%) before subsequent experiments. Non-ionic Tergitol type 15-S-12 (T15S12) secondary alcohol ethoxylated (SAE)-based biodegradable surfactant (CAS No.: 84133-50-6) (Figure 1) was purchased from Sigma Aldrich, St Louis, MO, USA. Octane (98+% purity) (CAS No.: 111-65-9) and n-octanol (>99% purity) (CAS No.: 111-87-5) were purchased from Alfa Aesar. They were of reagent grade. Sodium chloride (NaCl) (CAS No.: 7647-14-5) was purchased from VWR and used in a conventional water-based dyeing system only. Sodium carbonate anhydrous (soda ash, Na_2_CO_3_) (CAS No.: 497-19-8) was purchased from Sigma Aldrich. Warm-type reactive dyes of Levafix Red CA (RCA), Levafix Blue CA (BCA) and Levafix Yellow CA (YCA) were purchased from Dystar, China, and used as received.

### 2.2. Conventional Water Dyeing of Cotton Fabric

**Figure 2:**
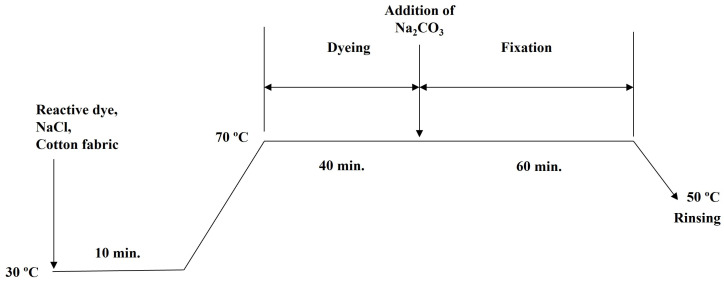
Dyeing workflow of cotton fabric in water (NaCl and Na_2_CO_3_).

Cotton fabric was conventionally dyed in water medium with the use of NaCl and Na_2_CO_3_ as auxiliaries to promote exhaustion and fixation. The recipe was used as recommended by the dye supplier (Table 1). The liquor-to-goods ratio was 50:1 to ensure the levelness of the dyed fabric. Cotton fabric was dyed according to the profile shown in Figure 2. Cotton fabric was immersed in dye liquor composed of a relative amount of reactive dye and placed in a water bath (30 °C) for 10 min shaking. The temperature of the bath was raised to 70 °C to allow dyeing for 40 min. Na_2_CO_3_ was then added to the dye liquor to allow fixation for 60 min. The dyed fabric was finally rinsed with 2 g/L of detergent twice (50 °C), cold-rinsed with tap water, air-dried and conditioned in standard environment (20 ± 2) °C and (65 ± 2) % for 24 h before further measurements.

### 2.3. SAE-Based Reverse Micellar Dye Carrier Formation

**Figure 3:**
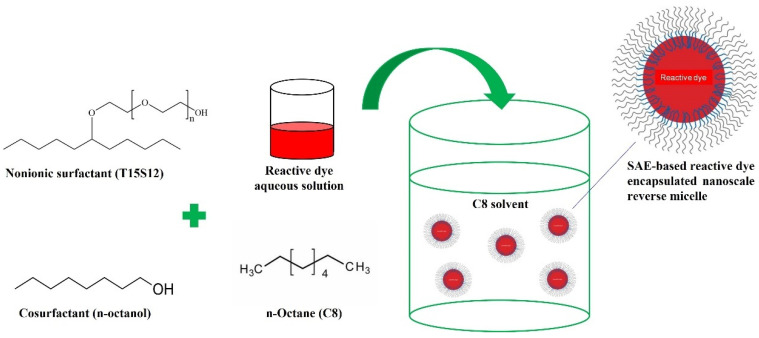
Preparation of SAE-based dye-encapsulated reverse micelles in octane.

Figure 3 shows the workflow of an SAE-based dye-encapsulated reverse micelle formation. T15S12 biodegradable surfactant was mixed with n-octanol co-surfactant to form a mixture. Octane, acted as a nonpolar solvent phase, was then added to the mixture and stirred continuously to facilitate the self-assembly of SAE-based reverse micelles at room temperature. Reactive dye aqueous solution was then added dropwise into the reverse micellar solution with stirring to reinforce carrier formation by encapsulating reactive dye in the interior water-pool region of the SAE-based reverse micelles.

### 2.4. Optimisation of Parameters for SAE-Based Reverse Micellar Dyeing System

A dye concentration of 3.5% o.w.f. Levafix Red CA reactive dye was used to optimise several parameters of the SAE-based reverse micellar dyeing system. A one-factor-at-a-time approach was used for optimising the parameters. In this approach, in order to study the influence of one factor, all other factors are kept constant. The parameters involved: (i) effect of colour fixation agent; (ii) surfactant-to-water mole ratio (1:20, 1:25, 1:30, 1:40 and 1:50); (iii) surfactant-to-co-surfactant mole ratio (1:6, 1:8, 1:10, 1:12, 1:15 and 1:20); (iv) volume of soda ash (0, 0.3, 0.4, 0.5, 0.6 and 0.7 mL); (v) volume of dye (0.3, 0.4, 0.5, 0.6 and 0.7 mL); (vi) solvent-to-cotton ratio (*v*/*w*) (8:1, 10:1, 12:1, 15:1 and 20:1); (vii) dyeing temperature (50, 60, 70, and 80 °C); (viii) dyeing time (10, 20, 30, 40, and 50 min); (ix) fixation time (10, 20, 30, 40, 50 and 60 min); (x) soda-ash-to-cotton ratio (g/g). The optimised parameters were then applied for dyeing of cotton fabrics in five colour depths on weight of fibre (o.w.f.) (0.1, 0.5, 1.5, 2.5 and 3.5%) with the use of BCA, RCA and YCA reactive dyes.

### 2.5. SAE-Based Salt-Free Reverse Micellar Dyeing of Cotton Fabric

**Figure 4:**
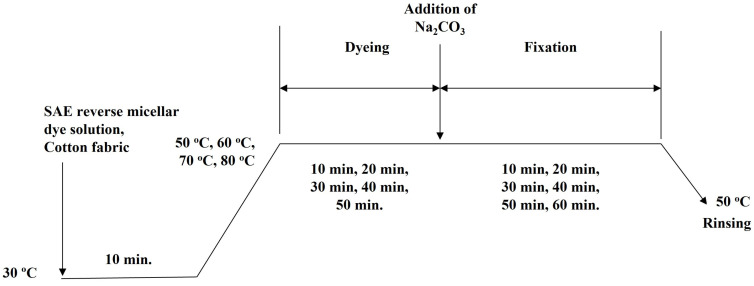
Salt-free reverse micellar dyeing workflow of cotton fabric in octane.

Figure 4 shows the workflow of SAE-based reverse micellar dyeing of cotton without salt. Cotton fabric was immersed in SAE reverse micellar dye solution and placed into a water bath (30 °C) for 10 min shaking. The temperature of the water bath was then raised to different temperatures (50, 60, 70, and 80 °C) for different time durations (10, 20, 30, 40, and 50 min). After that, a fixation agent, Na_2_CO_3_, was added into the dye solution for colour fixation with different time durations (10, 20, 30, 40, 50 and 60 min). The dyed fabric was finally rinsed with 2 g/L of detergent twice (50 °C), cold-rinsed with tap water, air-dried and conditioned in standard environment (20 ± 2 °C and 65 ± 2%) for 24 h before further measurements.

### 2.6. Colour Strength (K/S_sum_ Value)

A DataColor SF650 Spectrophotometer (DataColor International, USA) was used to measure the colour strength (K/S_sum_ value) of water-dyed and SAE-dyed fabric samples throughout the visible spectrum of 400 to 700 nm. The parameters for measurement were stated as follows: (a) aperture of 20 mm diameters; (b) illuminant D_65_; (c) 10° standard observer; (d) opacity of fabric sample guaranteed by folding the fabric twice; € measurement only on the face side of fabric; (f) data collected at every 10 nm interval throughout the visible spectrum of 400 to 700 nm; and (g) average value of four measurements per sample. The K/S value of each sample was then calculated by Equation (1).

K/S = (1 − R)^2^/2R (1)

where K: absorption coefficient; S: scattering coefficient; and R: reflectance.

### 2.7. CIE L*a*b* Value Measurement

The CIE L*a*b* value of water-dyed and SAE-dyed fabric samples was measured using the same apparatus and parameters as stated in the colour strength section.

### 2.8. Levelness Evaluation

The colour levelness of water-dyed and SAE-dyed fabric samples was evaluated by using the Relative Unlevelness Indices suggested by Chong et al. [27]. Four spots of each sample were randomly selected and evaluated by using the same apparatus and parameters as mentioned in the colour strength section. Equation (2) was then used to calculate the RUI value of each sample. Generally speaking, the lower the RUI value of the sample, the better the colour levelness of the fabric sample.

(2) $$RUI=\sum_{\lambda =400}^{700}{(s}_{\lambda }/\bar{R})V_{\lambda }$$

where $s_{\lambda }$: standard deviation of reflectance value (specified wavelength); $\bar{R}$: reflectance value (specific wavelength); $V_{\lambda }$: photopic relative luminous efficiency function.

### 2.9. Scanning Electron Microscopy (SEM)

An Hitachi VP-SEM SU1510 scanning electron microscope (Hitachi, Tokyo, Japan) was used to assess the fibre surface properties and damage of water-dyed and SAE-dyed cotton samples.

### 2.10. Transmission Electron Microscopy (TEM)

A JEM 2010 transmission electron microscope (JEOL Co. Tokyo, Japan) with 120 kV accelerating voltage and 69 mA beam current was used to examine the dye assembly morphology in the reverse micelles.

### 2.11. Fastness Properties Tests

AATCC Test Method 61-2013, Test No. 2A, was performed to examine the washing fastness (colour change and colour staining) of the dyed cotton samples and the attached multifibre adjacent fabrics. AATCC Test Method 8-2013 was conducted to assess the crocking fastness (colour staining) of the dyed cotton samples and the white cloth. The colourfastness to perspiration of dyed fabrics was evaluated according to AATCC Test Method 15-2013 (Colorfastness to Perspiration). The colourfastness to light of dyed fabrics was assessed according to AATCC Test Method 16-2013 (Colorfastness to Light: Xenon Arc) by using light fastness tester (Xenotest 440, ATLAS, Hamburg, Germany). The rating of these fastness tests was given by using the grey scale.

### 2.12. Tensile Properties

The ASTM D5034 standard (Breaking Strength and Elongation of Textile Fabrics: Grab Test) was performed to evaluate the breaking strength and extension of both water-dyed and SAE-dyed samples prepared at 3.5% o.w.f. dye concentration.

## 3. Results and Discussion

### 3.1. Effect of Colour Fixation Agent (Soda Ash)

**Figure 5:**
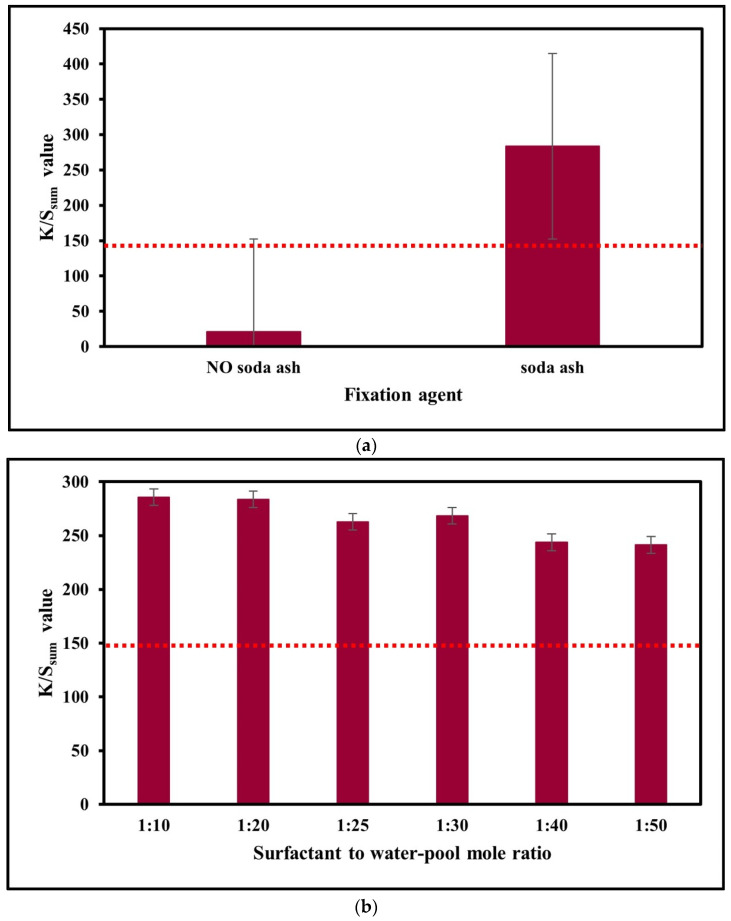

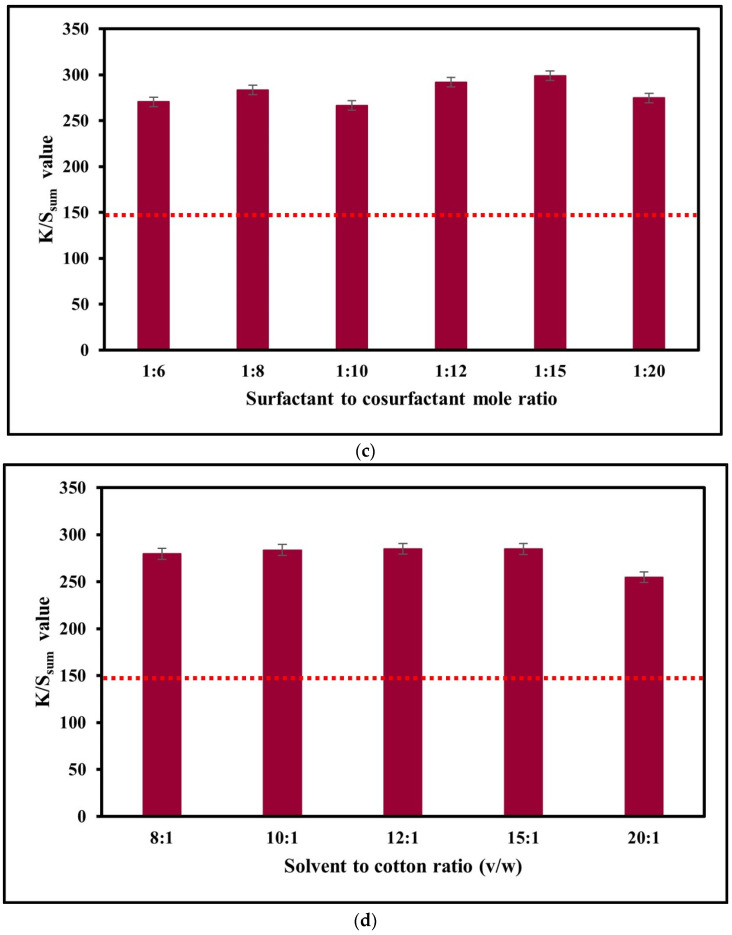

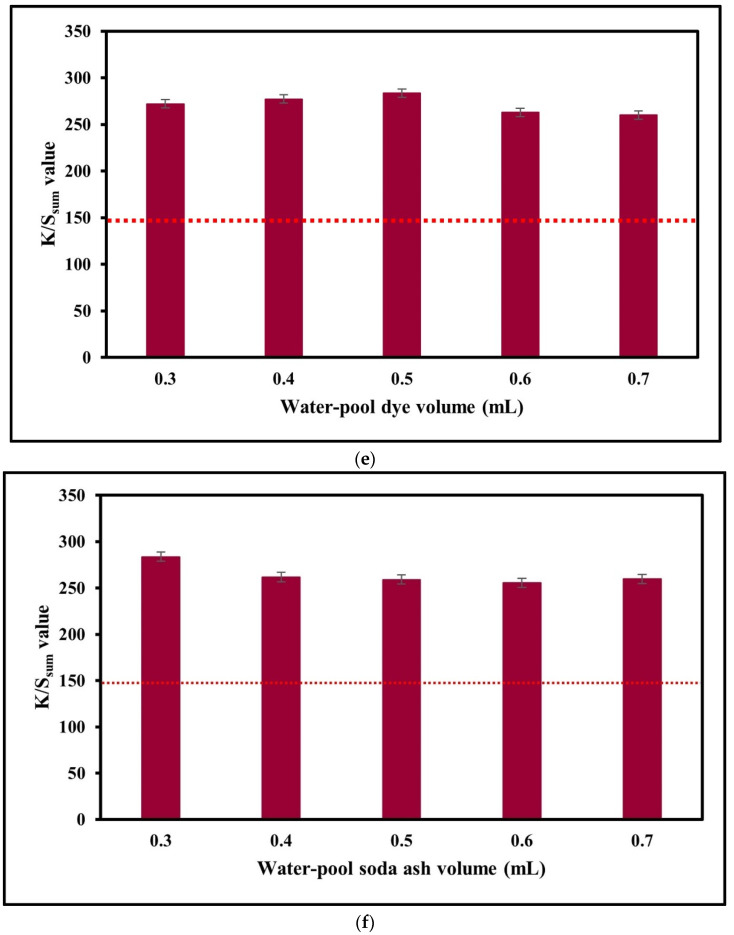

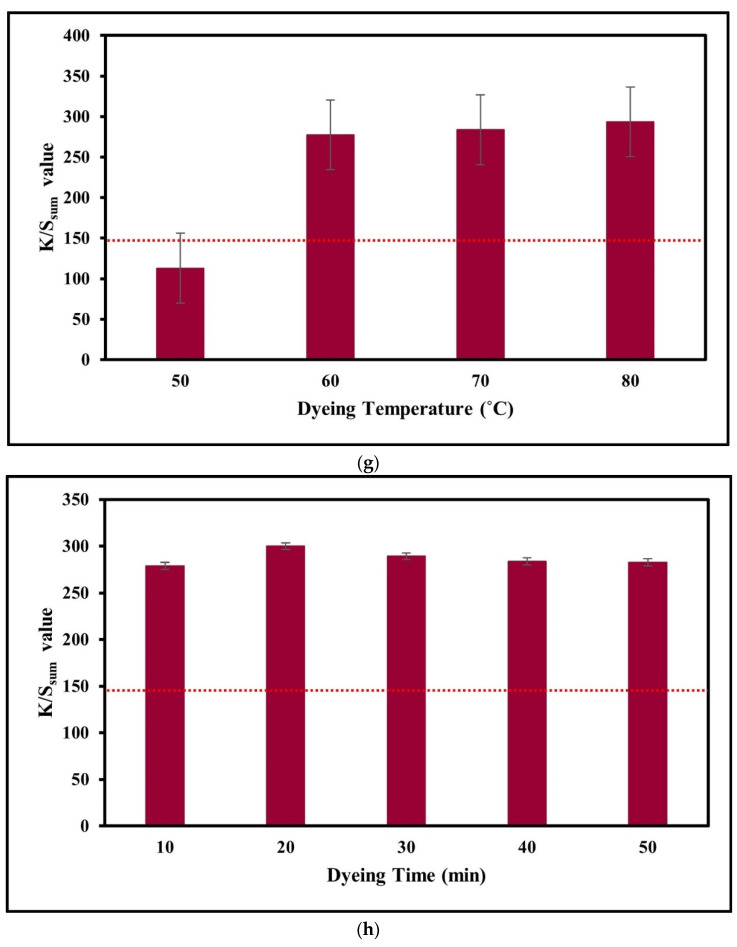

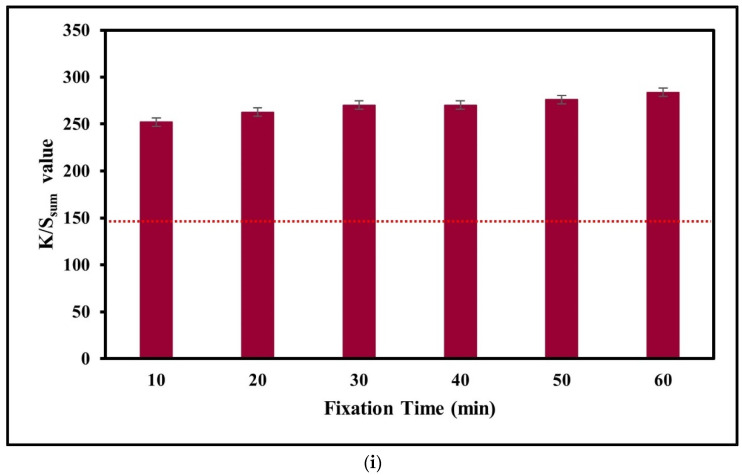
(**a**) Effect of colour fixation agent on colour strength (K/S_sum_ value) of SAE-dyed cotton fabric samples (dyeing parameters: surfactant-to-water-pool mole ratio: 1:20; surfactant-to-co-surfactant mole ratio: 1:8; solvent-to-cotton (*v*/*w*) ratio: 10:1; dye volume: 0.5 mL; soda ash volume: 0.3 mL; dyeing temperature: 70 °C; dyeing time: 40 min; fixation time: 60 min; soda-ash-to-cotton weight ratio: 0.07 g/g; dye concentration: 3.5% o.w.f.). The red horizontal dotted line represents the K/S_sum_ value of water-dyed fabric with a dye concentration of 3.5% o.w.f. (**b**) Effect of surfactant-to-water-pool mole ratio on colour strength (K/S_sum_ value) of SAE-dyed cotton fabric samples (dyeing parameters: surfactant-to-co-surfactant mole ratio: 1:8; solvent-to-cotton (*v*/*w*) ratio: 10:1; dye volume: 0.5 mL; soda ash volume: 0.3 mL; dyeing temperature: 70 °C; dyeing time: 40 min; fixation time: 60 min; soda-ash-to-cotton weight ratio: 0.07 g/g; dye concentration: 3.5% o.w.f.). The red horizontal dotted line represents the K/S_sum_ value of water-dyed fabric with a dye concentration of 3.5% o.w.f. (**c**) Effect of surfactant-to-co-surfactant mole ratio on colour strength (K/S_sum_ value) of SAE-dyed cotton fabric samples (dyeing parameters: surfactant-to-water-pool mole ratio: 1:20; solvent-to-cotton (*v*/*w*) ratio: 10:1; dye volume: 0.5 mL; soda ash volume: 0.3 mL; dyeing temperature: 70 °C; dyeing time: 40 min; fixation time: 60 min; soda-ash-to-cotton weight ratio: 0.07 g/g; dye concentration: 3.5% o.w.f.). The red horizontal dotted line represents the K/S_sum_ value of water-dyed fabric with a dye concentration of 3.5% o.w.f. (**d**) Effect of solvent-to-cotton ratio (*v*/*w*) on colour strength (K/S_sum_ value) of SAE-dyed cotton fabric samples (dyeing parameters: surfactant-to-water-pool mole ratio: 1:20; surfactant-to-co-surfactant mole ratio: 1:8; dye volume: 0.5 mL; soda ash volume: 0.3 mL; dyeing temperature: 70 °C; dyeing time: 40 min; fixation time: 60 min; soda-ash-to-cotton weight ratio: 0.07 g/g; dye concentration: 3.5% o.w.f.). The red horizontal dotted line represents the K/S_sum_ value of water-dyed fabric with a dye concentration of 3.5% o.w.f. (**e**). Effect of water-pool dye volume on colour strength (K/S_sum_ value) of SAE-dyed cotton fabric samples (dyeing parameters: surfactant-to-water-pool mole ratio: 1:20; surfactant-to-co-surfactant mole ratio: 1:8; solvent-to-cotton (*v*/*w*) ratio: 10:1; soda ash volume: 0.3 mL; dyeing temperature: 70 °C; dyeing time: 40 min; fixation time: 60 min; soda-ash-to-cotton weight ratio: 0.07 g/g; dye concentration: 3.5% o.w.f.). The red horizontal dotted line represents the K/S_sum_ value of water-dyed fabric with a dye concentration of 3.5% o.w.f. (**f**) Effect of water-pool soda ash volume on colour strength (K/S_sum_ value) of SAE-dyed cotton fabric samples (dyeing parameters: surfactant-to-water-pool mole ratio: 1:20; surfactant-to-co-surfactant mole ratio: 1:8; solvent-to-cotton (*v*/*w*) ratio: 10:1; dye volume: 0.5 mL; dyeing temperature: 70 °C; dyeing time: 40 min; fixation time: 60 min; soda-ash-to-cotton weight ratio: 0.07 g/g; dye concentration: 3.5% o.w.f.). The red horizontal dotted line represents the K/S_sum_ value of water-dyed fabric with a dye concentration of 3.5% o.w.f. (**g**) Effect of dyeing temperature on colour strength (K/S_sum_ value) of SAE-dyed cotton fabric samples (dyeing parameters: surfactant-to-water-pool mole ratio: 1:20; surfactant-to-co-surfactant mole ratio: 1:8; solvent-to-cotton (*v*/*w*) ratio: 10:1; dye volume: 0.5 mL; soda ash volume: 0.3 mL; dyeing time: 40 min; fixation time: 60 min; soda-ash-to-cotton weight ratio: 0.07 g/g; dye concentration: 3.5% o.w.f.). The red horizontal dotted line represents the K/S_sum_ value of water-dyed fabric with a dye concentration of 3.5% o.w.f. (**h**) Effect of dyeing time on colour strength (K/S_sum_ value) of SAE-dyed cotton fabric samples (dyeing parameters: surfactant-to-water-pool mole ratio: 1:20; surfactant-to-co-surfactant mole ratio: 1:8; solvent-to-cotton (*v*/*w*) ratio: 10:1; dye volume: 0.5 mL; soda ash volume: 0.3 mL; dyeing temperature: 70 °C; fixation time: 60 min; soda-ash-to-cotton weight ratio: 0.07 g/g; dye concentration: 3.5% o.w.f.). The red horizontal dotted line represents the K/S_sum_ value of water-dyed fabric with a dye concentration of 3.5% o.w.f. (**i**) Effect of fixation time on colour strength (K/S_sum_ value) of SAE-dyed cotton fabric samples (dyeing parameters: surfactant-to-water-pool mole ratio: 1:20; surfactant-to-co-surfactant mole ratio: 1:8; solvent-to-cotton (*v*/*w*) ratio: 10:1; dye volume: 0.5 mL; soda ash volume: 0.3 mL; dyeing temperature: 70 °C; dyeing time: 40 min; soda-ash-to-cotton weight ratio: 0.07 g/g; dye concentration: 3.5% o.w.f.). The red horizontal dotted line represents the K/S_sum_ value of water-dyed fabric with a dye concentration of 3.5% o.w.f.

Sodium carbonate (soda ash, Na_2_CO_3_) is one of the alkalis commonly used for coloration of cotton fabric with reactive dyes. It basically plays a role as a colour fixation agent in a conventional water-based dyeing system to promote chemical bonding and interaction between reactive dye and cotton fibre in alkaline condition. Figure 5a exhibits the colour strength (K/S_sum_ value) of SAE-dyed cotton samples with and without the addition of a colour fixation agent. Unlike a rhamnolipid biosurfactant-based reverse micellar dyeing system which can achieve similar colour yield in the absence of a colour fixation agent [24], the experiment result reveals that the colour strength of the SAE-dyed sample in the presence of soda ash as a fixation agent (colour strength: 283.6) is about 13.5 times higher than that of the sample without the influence of soda ash (colour strength: 21.4). In other words, an SAE-based reverse micellar dyeing system, similar to PEG-based [22] and APG-based systems [23], requires alkali (soda ash) to promote colour fixation of dye molecules into the fibre matrix.

### 3.2. Surfactant-to-Water-Pool Mole Ratio (SW Ratio)

Surfactant serves as the building block for the formation of reverse micelles [28,29], and it is one of the crucial factors affecting the SAE salt-free dyeing system. Figure 5b shows the effect of the SW ratio on the colour strength (K/S_sum_ value) of SAE-dyed cotton samples. Generally speaking, the colour strength of the dyed sample demonstrates a decreasing trend when the SW ratio increases from 1:10 to 1:50. The highest K/S_sum_ value (about 285) is found at an SW ratio of 1:10, while the lowest K/S_sum_ value (about 241) is obtained when an SW ratio of 1:50 is used. It means that the use of more surfactant contributes to higher colour strength of the SAE-dyed fabrics when the amount of water in the water-pool region remains constant, since a higher amount of surfactant contributes better dye encapsulation stability in reverse micelle [24,28]. However, it does not mean that an SW ratio of 1:10 is the best choice, since a 1:10 mole ratio needs to use double the amount of surfactant without attributing much increase to the colour strength of the dyed sample when compared with that of a 1:20 mole ratio (about 284). Lower SW ratios, such as 1:40 and 1:50, may result in a higher risk of fabric colour unlevelness, since less surfactant amount may lead to the reduction of total dye liquor volume in which the fabric sample may not be fully immersed in the dye liquor. Considering the use of more surfactant (1:10 SW ratio) may lead to an increase in the dyeing cost; 1:20 is thus recommended as the optimum SW ratio in SAE salt-free dyeing of cotton fabric.

### 3.3. Surfactant-to-Co-Surfactant Mole Ratio (SC Ratio)

The amount of co-surfactant, in relation to that of surfactant, is of great importance in the SAE salt-free dyeing system, since co-surfactant facilitates the self-assembly of reverse micelles and affects the interfacial curvature of water-pool partition. As presented in Figure 5c, the colour strength (K/S_sum_ value) of the SAE-dyed cotton samples fluctuates when different SC ratios are used. The sample dyed by an SC ratio of 1:15 obtains the highest colour strength (about 299), followed by the 1:12 and 1:8 SC ratios, while the sample dyed by a 1:10 SC ratio reveals the lowest colour strength (about 266). The use of too high (1:20) or too low (1:6) an SC ratio also results in poorer colour strength, since an optimum amount of co-surfactant is essential to maintain dye encapsulation and interfacial stability of the reverse micelle and to control the distribution of dye on the fibre surface [28]. Although SC ratios of 1:15 and 1:12 can gain comparatively higher colour strength, it is observed that the colour levelness of the samples is relatively poorer than the SC ratio of 1:8. With the increase in cost owing to the increase in co-surfactant amount, it is highly suggested that the optimum SC ratio in an SAE salt-free reverse micellar dyeing system should be 1:8.

### 3.4. Solvent-Volume-to-Cotton-Weight Ratio (Solvent Ratio)

Solvent, basically nonpolar in nature, acts as a non-aqueous dyeing medium in an SAE-based reverse micellar dyeing system. Figure 5d represents the effect of solvent ratio on the colour strength of the SAE-dyed fabric samples. A slight rising trend is generally observed on the K/S_sum_ value of the dyed samples when the solvent ratio increases from 8:1 (about 280) to 12:1 (about 285). The colour strength of the dyed samples falls slightly (about 284) when a 15:1 solvent ratio is used. However, an obvious downfall of colour strength is noted when samples are dyed with a 20:1 solvent ratio (about 255). The decrease in colour strength at a high solvent ratio is possibly the result of dye aggregation, in which the size of reverse micelles becomes too bulky for dye diffusion in the fibre matrix, leading to relatively lower dye uptake [28]. As the colour strength does not rise too much between 8:1 and 12:1, it is recommended that the optimum solvent ratio should be 10:1, so as to strike a balance between the cost and the risk of incomplete immersion of fabric sample in solvent medium.

### 3.5. Dye Volume in Water-Pool Region

The colour strength (K/S_sum_ value) of SAE-dyed samples with the use of different dye volumes in the water-pool region of the reverse micelle is illustrated in Figure 5e. The K/S_sum_ value rises from 272 and reaches the highest value of 283.6 when the dye volume in the water pool increases from 0.3 mL to 0.5 mL. A further increase in dye volume to 0.6 and 0.7 mL only results in a decrease in K/S_sum_ value (262 and 260, respectively). Too low or too high a volume of dye in the water pool may affect its solubilisation and dispersion in reverse micelle, leading to the formation of undesired dye aggregates [24,28]. Therefore, it is believed that 0.5 mL would be the optimum water-pool dye volume in an SAE-based reverse micellar system.

### 3.6. Soda Ash Volume in Water-Pool Region

Figure 5f demonstrates the colour strength (K/S_sum_ value) of SAE-dyed cotton samples with different soda ash volumes in the water-pool region of reverse micelles. The colour strength generally exhibits a declining trend from 283.6 to 255.6 with increasing soda ash volume from 0.3 mL to 0.6 mL, whereas the colour strength shows a slight increase to 260 when 0.7 mL of soda ash volume is used. This suggests that the optimum volume for effective solubilisation of soda ash in the water-pool region of reverse micelles should be kept in a minimum of 0.3 mL. A further increase in soda ash volume not only lowers the colour strength of fabric samples but also increases the total cost of the SAE-based reverse micellar system, since the increase in soda ash volume may enlarge the size of the reverse micelles, requiring higher amounts of surfactant and co-surfactant to maintain water-pool stability and well encapsulation.

### 3.7. Dyeing Temperature

The effect of dyeing temperature on the colour strength (K/S_sum_ value) of SAE-dyed samples is presented in Figure 5g. Generally speaking, the colour strength of the dyed fabric shows a rising trend in relation to the dyeing temperature. It rises from 112.8 to 293.4 when the dyeing temperature increases from 50 °C to 80 °C. Compared with those samples dyed at a higher temperature, fabric dyed at 50 °C results in a significantly lower colour strength. It may be the cause of the optimum reaction or working temperature of warm-type reactive dyes, which is normally above 60 °C. Although high temperature (80 °C) gains the highest K/S_sum_ value, it may have a higher risk of thermal degradation of reactive dye and may increase the energy cost without significantly increasing the colour strength of the dyed samples. Therefore, it is suggested that the dyeing temperature should be maintained at 70 °C for dyeing cotton with warm-type reactive dyes.

### 3.8. Dyeing Time

The colour strength of an SAE-based reverse micellar-dyed cotton sample under different dyeing times is displayed in Figure 5h. It can be seen that the colour strength of the SAE-dyed sample increases from 278.8 to 299.9 when the dyeing time increases from 10 min to 20 min. The colour strength of the dyed fabrics then reveals a declining trend from 299.9 to 282.5 (50 min). It seems that a dyeing time of 20 min is the best choice in terms of colour strength. However, the dyeing process involves both absorption and desorption mechanisms. It requires sufficient time to reach the dyeing equilibrium in order to achieve better dye migration, distribution and diffusion into the fibre with minimal dye aggregation and colour unlevelness. Therefore, it would be better to select 40 min as the recommended dyeing time, since the difference in colour strength of the dyed fabric between 40 min and 50 min is minimal (less than 1), which denotes the equilibrium dyeing time with relatively stable colour strength of the dyed fabric.

### 3.9. Fixation Time

Figure 5i shows the colour strength (K/S_sum_ value) of SAE-dyed samples at different fixation times. Generally speaking, the colour strength of the dyed samples rises steadily from 251.9 to 283.6 when the fixation time increases from 10 min to 60 min. This indicates that a short fixation time, such as 10 min, is insufficient for dye fixation into the fibre matrix. A longer fixation time is needed in order to obtain the optimum colour strength and reduce dye residuals remaining in the dye liquor, causing the wastage of dyestuff and the production of more effluent. Therefore, a fixation time of 60 min is recommended to optimise the dye exhaustion with minimised dye and auxiliary wastage.

### 3.10. Soda Ash Concentration

Since an SAE non-ionic surfactant-based reverse micellar dyeing system requires a colour fixation agent (soda ash) to promote the fixation of dye molecules in fibre and boost the colour strength of the dyed cotton fabric samples, as proved in Section 3.1., the soda ash concentration then becomes one of the important dyeing parameters in the system. As depicted in Table 2, three soda ash concentrations are selected for optimisation in each dye concentration (RCA 0.1 to 3.5%). The results reveal that the increase in soda ash concentration may not lead to an increase in colour strength (K/S_sum_ value) of the dyed samples. The colour strength of the dyed samples is similar (less than 10), especially when low dye concentrations (0.1 and 0.5%) are used. In the case of 0.5 and 3.5% dye concentrations, the highest colour strength of the dyed sample is found at low soda ash concentrations (0.04 and 0.07 g/g). The similar colour strength of the dyed samples in three soda ash concentrations may be due to the cause of the pH value and the colour fixation agent. Further investigation will be conducted in our next work based on this finding.

### 3.11. Optimum Parameters for SAE-Based Reverse Micellar Dyeing of Cotton

The optimum dyeing parameters for SAE-based reverse micellar dyeing of cotton fabric with red reactive dye (RCA) are summarised in Table 3. These parameters are then directly applied for dyeing of cotton fabric with blue and yellow reactive dyes (BCA and YCA).

### 3.12. Colour Strength

The colour strength (K/S_sum_ value) of water-dyed and SAE-dyed cotton samples with three reactive dyes (BCA, RCA and YCA) is presented in Table 4. SAE-dyed samples generally can achieve higher colour strength than that of water-dyed samples. It may be the result of reduced ionisation effect between the dye and fibre and improved fibre swelling in a non-aqueous solvent-assisted SAE-based reverse micellar dyeing system [18,28]. Samples dyed by a T15S12 SAE biodegradable surfactant-based reverse micellar system with BCA reactive dye can obtain 31.4 to 48.3% higher colour strength than that of samples dyed by a conventional water-based system. The highest percentage increase in colour strength is found (between 97.1 and 145.2%) when samples are dyed by an SAE reverse micellar system with RCA reactive dye. The substantial increase in colour strength of the SAE-dyed samples may be due to the optimisation effort and the compatibility of RCA reactive dye [22,24]. In the case of YCA reactive dye, the colour strength of SAE-dyed samples, although the percentage increase in colour strength is the lowest among the three reactive dyes, is 20.1 to 27.3% higher than that of the water-dyed samples. This finding indicates that using a T15S12 SAE biodegradable surfactant-based reverse micellar system for dyeing of cotton fabric in non-aqueous octane medium is superior to that of the conventional water-based dyeing system. In addition, it is found that the standard deviation of the K/S_sum_ value of the dyed samples generally increases with increasing dye concentration. Compared with the samples dyed in the water-based system (0.02 to 5.48), the standard deviation of the K/S_sum_ value on SAE-dyed samples is slightly higher (0.11 to 5.73). However, the similarity is that samples dyed by BCA reactive dye in both water (0.05 to 2.65) and SAE reverse micellar systems (0.11 to 5.03) obtain the lowest standard deviation on K/S_sum_ value, followed by those dyed by YCA reactive dye (water: 0.15 to 4.78; SAE: 0.39 to 5.19), whereas samples dyed by RCA reactive dye attain the highest standard deviation on the K/S_sum_ value (water: 0.02 to 5.48; SAE: 0.22 to 5.73).

### 3.13. Reflectance

**Figure 6:**
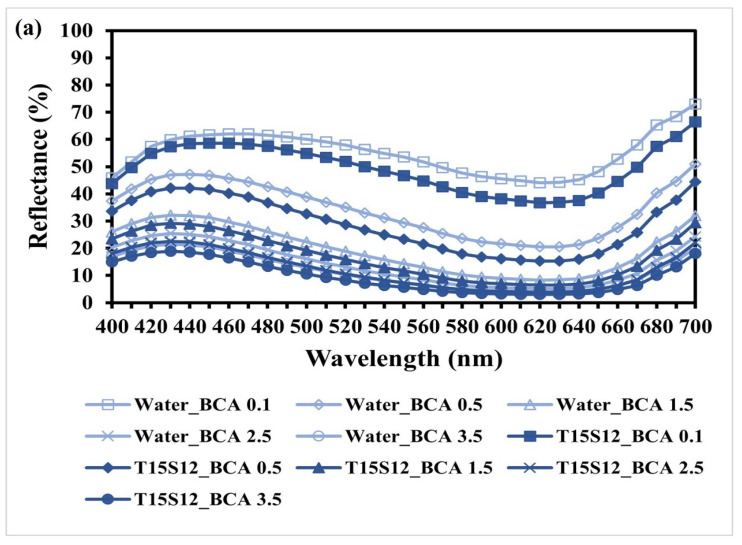

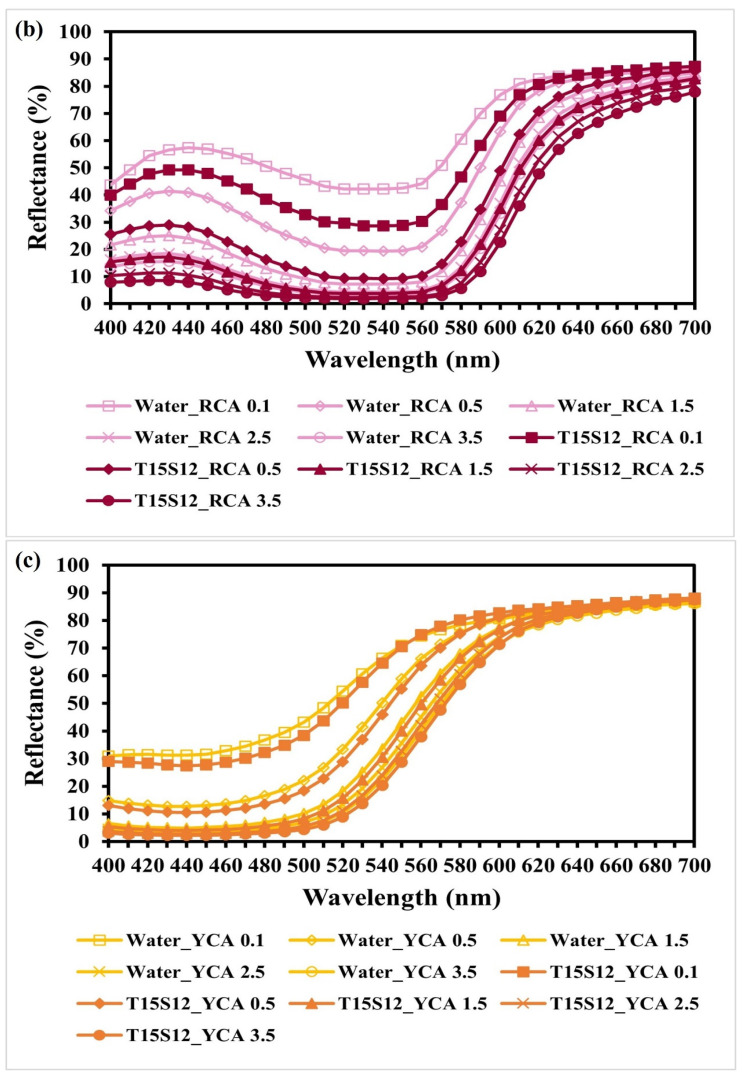
Reflectance curves of dyed cotton samples: (**a**) BCA; (**b**) RCA; and (**c**) YCA.

Figure 6a–c represent the reflectance curves of water-dyed and T15S12 SAE-dyed cotton fabrics with blue, red and yellow reactive dyes, respectively. It is observed that the reflectance percentage of water-dyed samples is generally higher than that of SAE-dyed samples. This indicates that the SAE-dyed samples generally possess a darker shade than the SAE-dyed samples, with lower reflectance and better dye absorption. In addition, the reflectance curves of SAE-dyed samples are identical in shape when compared with those of water-dyed samples. This confirms that an SAE-based reverse micellar dyeing system does not lead to peak shifting of the reflectance curves and chromatic change of the dyed samples, guaranteeing the colour quality of the SAE-dyed fabric samples.

### 3.14. Colour Levelness

**Figure 7:**
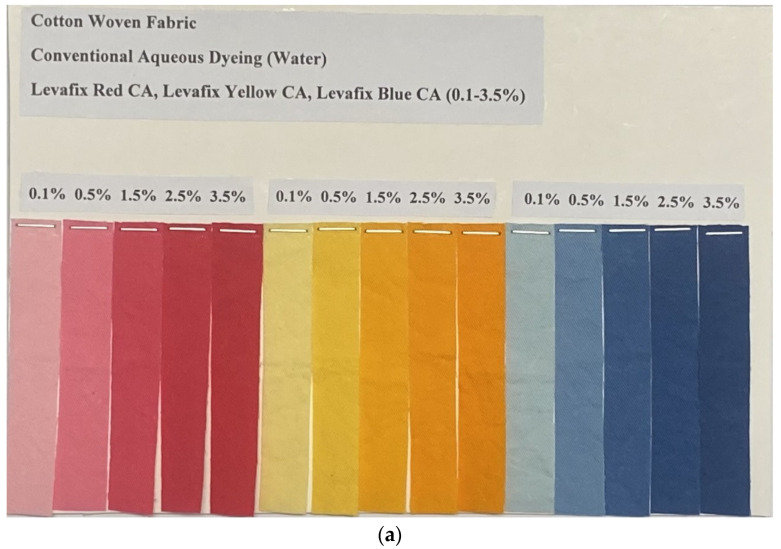

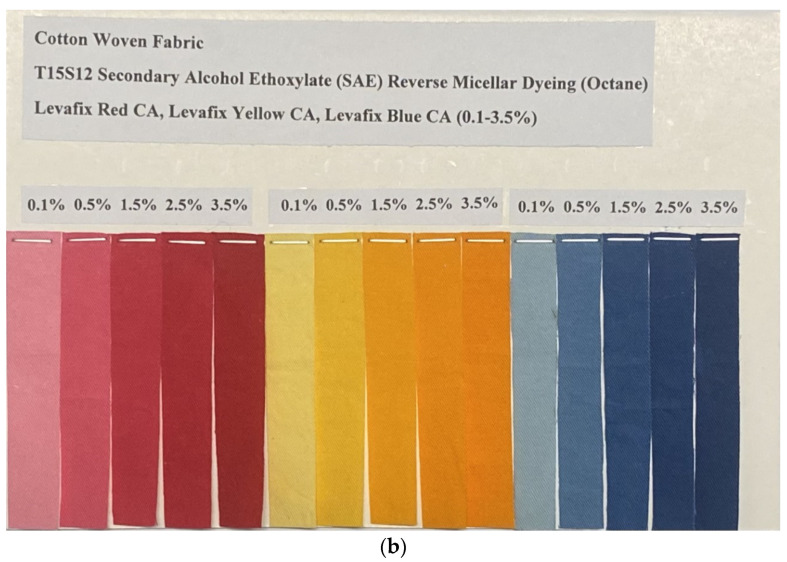
Visual images: (**a**) water-dyed samples; (**b**) T15S12 SAE-dyed samples.

Table 5 and Figure 7 demonstrate the RUI values and the visual images of water-dyed and T15S12 SAE-dyed cotton samples. It is found that both water-dyed and SAE-dyed samples obtain good-to-excellent colour levelness, with the RUI values range from 0.03 to 0.27 and between 0.04 and 0.46, respectively. Among the three reactive dyes, samples dyed with yellow reactive dye, including both water-dyed and SAE-dyed samples, gain the best colour levelness (RUI value: 0.03 to 0.12 and 0.05 to 0.15). Good-to-excellent levelness of water-dyed samples and SAE-dyed samples is possibly attributed to the use of a 50:1 liquor ratio and the optimisation work, respectively. This validates that a T15S12 SAE biodegradable surfactant-based reverse micellar dyeing system, after the optimisation of dyeing parameters, can achieve good-to-excellent levelness, which is comparable to a conventional water-based dyeing system.

### 3.15. CIE L*a*b* Value

The CIE L*a*b* values of cotton samples dyed in a conventional water-based system and a T15S12 SAE-based reverse micellar system are illustrated in Table 6. With regard to BCA reactive dye, SAE-dyed samples generally acquire a lower L* value and b* value than water-dyed samples. This indicates that SAE-dyed samples, compared with water-dyed samples, are darker and bluer in shade. Concerning RCA reactive dye, SAE-dyed samples obtain lower L*, and higher a* and b* values than water-dyed samples. This means that SAE-dyed samples are darker, redder and yellower in shade in comparison to water-dyed samples. In the case of YCA reactive dye, SAE-dyed samples obtain slightly lower L*, higher and b* values than water-dyed samples. This represents that SAE-dyed samples are darker, redder and yellower in shade when compared with water-dyed samples when yellow reactive dye is used.

### 3.16. SEM Images

**Figure 8:**
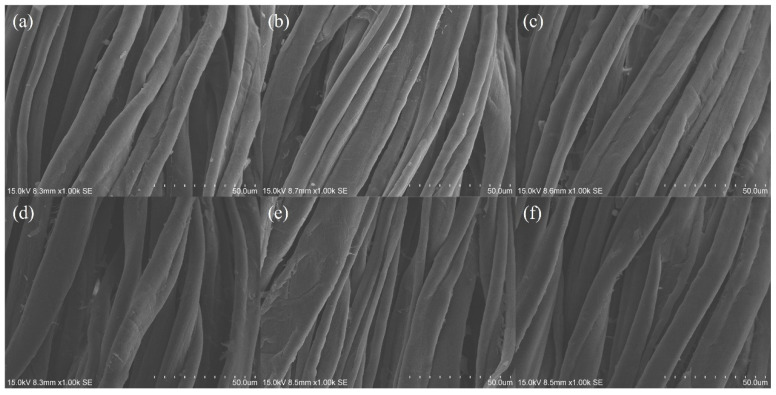
SEM images of water-dyed and SAE-dyed cotton fabric samples (magnification: 1000×): (**a**) Water–BCA; (**b**) Water–RCA; (**c**) Water–YCA; (**d**) SAE–BCA; (**e**) SAE–RCA; and (**f**) SAE–YCA.

The fibre surface morphology of water-dyed and SAE-dyed cotton fabric samples is shown in Figure 8. Both the conventional water-based dyeing approach and the SAE salt-free reverse micellar dyeing approach do not cause significant damage on the surface of the cotton fibre after the dyeing process. This validates that the SAE-based salt-free reverse micellar dyeing approach is feasible for dyeing of cotton fabric in non-aqueous octane medium without causing severe damage on the fibre surface.

### 3.17. TEM Image

**Figure 9:**
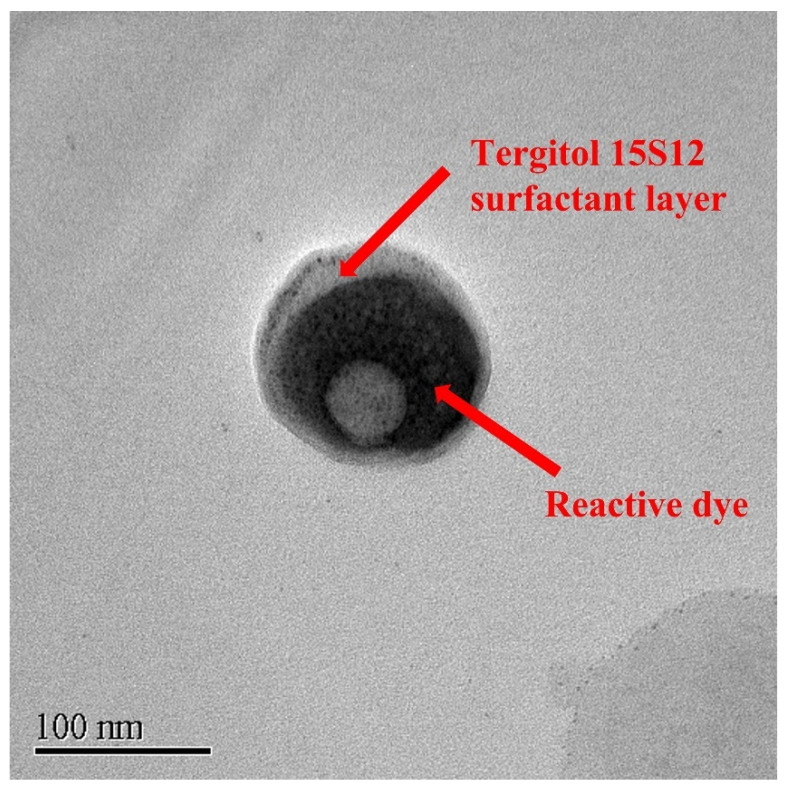
TEM image of red reactive dye (RCA 1.5%)-encapsulated Tergitol 15S12 non-ionic surfactant-based reverse micelle (magnification: 40,000×).

Figure 9 exhibits the TEM image of the reactive-dye-encapsulated reverse micelle. The morphology of the reverse micelle reveals a spherical core–shell structure in which red reactive dye serves as the inner core (water pool) surrounded by the outer shell of the Tergitol type 15-S-12 (T15S12) non-ionic surfactant layer. The reverse micelle is of nanoscale with the diameter around 135 nm. The thickness of the outer surfactant layer ranges from 6 nm to 23 nm. The reactive dye is aesthetically encapsulated as circular or moon-shaped with the diameter around 100 to 112 nm. This finding validates that the cotton fabric samples are dyed in the presence of a spherical nanoscale dye-encapsulated reverse micelle formed by T15S12 non-ionic surfactant as the building block, serving as a reactive dye carrier in non-aqueous octane medium.

### 3.18. Washing and Crocking Fastness

Table 7 depicts the washing and crocking fastness of water-dyed and SAE-dyed samples in terms of colour change and colour staining. Both conventional water-dyed samples and SAE octane-dyed samples have excellent ratings (rating 4-5) against colour change after the dyeing process. In the case of colour staining, both water-dyed and octane-dyed samples can also gain good-to-excellent ratings (rating 4 to 4-5). This indicates that SAE reverse micellar octane-dyed samples can achieve excellent washing fastness which is comparable to that of conventional water-dyed samples.

As depicted in Table 7, both water-dyed samples and octane-dyed samples can attain good-to-excellent dry and wet crocking fastness (rating between 4 and 4-5). This confirms that SAE reverse micellar octane-dyed samples can also achieve dry and wet fastness comparable to that of conventional water-dyed samples.

### 3.19. Perspiration and Light Fastness

Table 8 presents the perspiration and light fastness of water-dyed and SAE octane-dyed samples. Generally speaking, water-dyed samples obtain an excellent rating of 4-5 on light fastness and perspiration fastness, whereas SAE reverse micellar dyed fabric samples can obtain good-to-excellent fastness against light and perspiration, with a rating between 4 and 4-5. This indicates that SAE reverse micellar dyed samples can achieve light and perspiration fastness which are comparable to that of the water-dyed samples, validating the feasibility of using SAE non-ionic surfactant-based reverse micelles as a dye carrier for dyeing of cotton fabric in octane medium.

### 3.20. Tensile Strength and Breaking Extension

The tensile strength values (N) of pristine, conventional water-dyed and SAE octane-dyed cotton samples in the warp and weft directions are listed in Table 9. Generally speaking, pristine cotton samples have the highest tensile strength (415 N), followed by octane-dyed samples (321–358 N) and water-dyed samples (317–346 N) in the warp direction. In the case of weft direction, octane-dyed samples (216–226 N) can achieve slightly higher tensile strength than pristine cotton samples (216 N), whereas water-dyed samples (209–215 N) have slightly lower tensile strength than undyed cotton samples (216 N). It is believed that the decrease in tensile strength was mainly due to the cause of temperature and pH value (acidity and alkalinity) during the dyeing process [30]. The length (mm) and percentage (%) of breaking extension of undyed, water-dyed and octane-dyed samples are depicted in Table 10. It is observed that the elongation at break along the warp direction is generally higher than that along the weft direction for both undyed, water-dyed and octane-dyed samples. Octane-dyed samples can achieve similar breaking extension to undyed and water-dyed cotton samples, indicating that SAE-based salt-free reverse micellar dyeing in octane medium does not severely alter the tensile properties of the dyed cotton fabrics.

## 4. Conclusions

This study aims at investigating the feasibility of using biodegradable Tergitol type 15-S-12 (T15S12) secondary alcohol ethoxylate (SAE) non-ionic surfactant as a building block for the formation of reverse micelles, functioning as reactive dye carriers for the dyeing of cotton fabric in non-aqueous octane medium. Ten dyeing parameters were optimised with the use of red reactive dye in terms of colour strength (K/S_sum_ value) of the dyed samples and directly applied to blue and yellow reactive dyes. The colour properties, fastness properties and physical properties of SAE-dyed samples were then compared with the conventional water-dyed samples.

Experimental results show that SAE-dyed samples have better colour strength and lower reflectance percentage than that of water-dyed samples. Reflectance curves are identical in shape without peak shifting and chromatic change between SAE-dyed samples and water-dyed samples. The CIE L*a*b* value was also measured. SAE-dyed samples generally obtain a darker shade with a lower L* value than that of water-dyed samples. The colour levelness of the dyed samples was evaluated. SAE-dyed samples can achieve good-to-excellent levelness comparable to water-dyed samples. These findings confirm that an SAE-based reverse micellar dyeing system is superior to a conventional water-based dyeing system.

Washing and crocking fastness of the dyed samples were evaluated. Both water-dyed and SAE-dyed samples can achieve excellent washing and crocking fastness, guaranteeing thorough removal of unfixed dye and auxiliaries and the accuracy of the obtained experimental results. The physical properties, in terms of breaking strength and extension, of the dyed samples were assessed. SAE-dyed samples can obtain similar breaking strength and extension to water-dyed samples. These findings ensure that SAE-dyed samples can achieve fastness and physical properties comparable to water-dyed samples.

SEM and TEM characterisation was conducted to observe the surface morphology of the dyed cotton fibre and the morphology of the reactive-dye-encapsulated reverse micelle, respectively. SEM images reveal that the dyed cotton fibres have no severe surface damage caused by an SAE-based reverse micellar dyeing system. The TEM image depicts that the reverse micelle is of nanoscale, spherical-shaped and has a core–shell structure, validating the presence of reverse micelle as a reactive dye carrier in an SAE-based reverse micellar dyeing system.

## Figures and Tables

**Table 1:** Recipe for dyeing of cotton in water.

Liquor Ratio 50:1, 70 °C						
Reactive dye	% o.w.f.	0.1	0.5	1.5	2.5	3.5
NaCl	g/L	10	20	42.5	55	65
Na_2_CO_3_	g/L	5	5	5	5	5

**Table 2:** Soda ash concentrations.

Dye Conc. (%)	Soda Ash to Cotton (g/g)	K/S_sum_ Value	Optimum Soda Ash Conc. (g/g)
RCA 0.1	0.07	11.76	0.03
	0.05	11.21	
	0.03	11.51	
RCA 0.5	0.08	46.50	0.04
	0.06	46.03	
	0.04	47.44	
RCA 1.5	0.09	137.85	0.05
	0.07	128.90	
	0.05	127.40	
RCA 2.5	0.10	239.86	0.06
	0.08	239.54	
	0.06	220.29	
RCA 3.5	0.12	271.94	0.07
	0.09	274.08	
	0.07	283.64	

**Table 3:** Optimised dyeing parameters in non-aqueous octane medium.

Optimised Dyeing Parameters					
Surfactant-to-water mole ratio			1:20		
Surfactant-to-co-surfactant mole ratio			1:8		
Solvent-to-cotton ratio (*v*/*w*)			10:1		
Water-pool volume for dye (mL)			0.5		
Water-pool volume for colour fixation agent (mL)			0.3		
Dyeing time (min)			40		
Fixation time (min)			60		
Dyeing and fixation temperature (°C)			70		
Dye concentration (% o.w.f.)	0.1	0.5	1.5	2.5	3.5
Soda-ash-to-cotton weight ratio (g/g)	0.03	0.04	0.05	0.06	0.07

**Table 4:** Colour strength of dyed cotton samples.

		Colour Strength (K/S_sum_ Value)				
Colour	Dye Conc.	Water	SD	SAE	SD	Increased in Colour Strength (%)
BCA	0.1%	6.19	0.1	9.01	0.1	45.6
	0.5%	23.57	0.2	34.96	1.2	48.3
	1.5%	71.35	1.2	93.77	1.3	31.4
	2.5%	121.10	3.2	161.80	2.1	33.6
	3.5%	163.17	2.7	216.79	5.0	32.9
RCA	0.1%	5.43	0.1	11.51	0.5	111.8
	0.5%	19.35	0.3	47.44	0.2	145.2
	1.5%	63.43	1.3	127.40	4.2	100.8
	2.5%	110.34	2.4	220.29	5.6	99.6
	3.5%	143.89	5.5	283.64	5.7	97.1
YCA	0.1%	8.25	0.2	10.21	0.4	23.7
	0.5%	30.07	0.6	38.26	0.8	27.2
	1.5%	88.90	1.6	111.73	5.3	25.7
	2.5%	137.97	0.7	171.19	3.2	24.1
	3.5%	177.10	4.8	212.80	5.2	20.2

Remark: SD = standard deviation.

**Table 5:** Relative Unlevelness Indices (RUI) of dyed cotton samples.

RUI					
Colour	Dye Conc.	Water	Visual	SAE	Visual
BCA	0.1%	0.03	Excellent	0.05	Excellent
	0.5%	0.05	Excellent	0.23	Good
	1.5%	0.13	Excellent	0.12	Excellent
	2.5%	0.24	Good	0.46	Good
	3.5%	0.15	Good	0.33	Good
RCA	0.1%	0.06	Excellent	0.12	Excellent
	0.5%	0.07	Excellent	0.04	Excellent
	1.5%	0.08	Excellent	0.26	Good
	2.5%	0.09	Good	0.20	Good
	3.5%	0.06	Good	0.29	Good
YCA	0.1%	0.03	Excellent	0.05	Excellent
	0.5%	0.06	Excellent	0.06	Excellent
	1.5%	0.09	Excellent	0.14	Excellent
	2.5%	0.05	Excellent	0.08	Excellent
	3.5%	0.12	Excellent	0.15	Excellent

Remark: less than 0.2 = excellent levelness; 0.2–0.49 = good levelness; 0.5–1.0 = poor levelness; more than 1.0 = bad levelness.

**Table 6:** CIE L*a*b* values of water-dyed and SAE-dyed cotton samples.

		Water			SAE		
Dye	Dye (%)	L*	a*	b*	L*	a*	b*
BCA	0.1	76.61	−6.87	−9.87	72.27	−8.33	−14.07
	0.5	59.29	−10.54	−23.18	53.55	−11.30	−26.67
	1.5	43.05	−12.02	−30.70	39.10	−12.12	−32.53
	2.5	35.46	−11.47	−32.63	31.53	−11.09	−34.00
	3.5	31.32	−10.59	−32.73	27.60	−9.79	−33.60
RCA	0.1	81.19	23.92	4.77	74.41	35.01	4.79
	0.5	69.94	41.61	7.28	61.46	51.00	11.61
	1.5	59.11	53.25	14.71	53.18	56.68	20.33
	2.5	54.44	56.32	19.59	48.63	57.70	26.74
	3.5	52.19	56.95	22.14	45.88	56.90	30.00
YCA	0.1	87.79	10.88	39.00	87.79	13.26	44.76
	0.5	83.79	21.39	65.61	82.97	24.48	70.76
	1.5	78.78	31.73	83.97	77.84	34.01	87.62
	2.5	75.99	35.96	88.84	75.07	38.34	91.76
	3.5	73.98	38.62	90.53	73.32	40.68	93.00

**Table 7:** Washing and crocking fastness of dyed cotton fabrics.

		Washing Fastness			Crocking Fastness	
	Dye Conc.	Colour Change	Colour Staining		Colour Staining	
	(%)	Rating	Wool	Cotton	Dry	Wet
BCA	0.1	4-5/4-5 *	4-5/4-5	4-5/4-5	4-5/4-5	4-5/4-5
	0.5	4-5/4-5	4-5/4-5	4-5/4-5	4-5/4-5	4-5/4-5
	1.5	4-5/4-5	4-5/4-5	4-5/4-5	4-5/4-5	4-5/4-5
	2.5	4-5/4-5	4-5/4-5	4-5/4	4-5/4	4-5/4
	3.5	4-5/4-5	4-5/4-5	4-5/4	4-5/4	4-5/4
RCA	0.1	4-5/4-5	4-5/4-5	5/5	4-5/4-5	4-5/4-5
	0.5	4-5/4-5	4-5/4-5	5/5	4-5/4-5	4-5/4-5
	1.5	4-5/4-5	4-5/4-5	5/5	4-5/4-5	4-5/4
	2.5	4-5/4-5	4-5/4-5	4-5/4	4-5/4	4-5/4
	3.5	4-5/4-5	4/4	4-5/4	4-5/4	4-5/4
YCA	0.1	4-5/4-5	4-5/4-5	4-5/4-5	4-5/4-5	4-5/4-5
	0.5	4-5/4-5	4-5/4-5	4-5/4-5	4-5/4-5	4-5/4-5
	1.5	4-5/4-5	4-5/4-5	4-5/4-5	4-5/4-5	4-5/4-5
	2.5	4-5/4-5	4-5/4-5	4-5/4-5	4-5/4-5	4-5/4
	3.5	4-5/4-5	4-5/4-5	4-5/4-5	4-5/4	4-5/4

Remark: 1 represents most colour change and staining; 5 represents least colour change and staining. * Rating indication: water-dyed sample/SAE reverse micellar octane-dyed sample.

**Table 8:** Perspiration and light fastness of dyed cotton fabrics.

		Light Fastness	Perspiration Fastness				
	Dye Conc.	Colour Change	Colour Change	Acidic Staining		Alkaline Staining	
	(%)	Rating	Rating	Wool	Cotton	Wool	Cotton
BCA	0.1	4-5/4-5 *	4-5/4-5	4-5/4-5	4-5/4-5	4-5/4-5	4-5/4-5
	0.5	4-5/4-5	4-5/4-5	4-5/4-5	4-5/4-5	4-5/4-5	4-5/4-5
	1.5	4-5/4-5	4-5/4-5	4-5/4-5	4-5/4-5	4-5/4-5	4-5/4
	2.5	4-5/4-5	4-5/4-5	4-5/4-5	4-5/4	4-5/4-5	4-5/4
	3.5	4-5/4	4-5/4-5	4-5/4-5	4-5/4	4-5/4-5	4-5/4
RCA	0.1	4-5/4-5	4-5/4-5	4-5/4-5	4-5/4-5	4-5/4-5	4-5
	0.5	4-5/4-5	4-5/4-5	4-5/4-5	4-5/4-5	4-5/4-5	4-5/4
	1.5	4-5/4	4-5/4-5	4-5/4-5	4-5/4-5	4-5/4-5	4-5/4
	2.5	4-5/4	4-5/4-5	4-5/4-5	4-5/4	4-5/4-5	4-5/4
	3.5	4-5/4	4-5/4-5	4-5/4-5	4-5/4	4-5/4-5	4-5/4
YCA	0.1	4-5/4-5	4-5/4-5	4-5/4-5	4-5/4-5	4-5/4-5	4-5
	0.5	4-5/4-5	4-5/4-5	4-5/4-5	4-5/4-5	4-5/4-5	4-5/4
	1.5	4-5/4-5	4-5/4-5	4-5/4-5	4-5/4-5	4-5/4-5	4-5/4
	2.5	4-5/4-5	4-5/4-5	4-5/4-5	4-5/4-5	4-5/4-5	4-5/4
	3.5	4-5/4-5	4-5/4-5	4-5/4-5	4-5/4-5	4-5/4-5	4-5/4

Remark: 1 represents most colour staining; 5 represents least colour staining. * Rating indication: water-dyed sample/SAE reverse micellar octane-dyed sample.

**Table 9:** Tensile strength of undyed, water-dyed and SAE-dyed cotton samples.

Tensile Strength (N)	Undyed		Water				SAE			
Sample	Warp	Weft	Warp	(%)	Weft	(%)	Warp	(%)	Weft	(%)
BCA 3.5	414.76	215.83	345.73	−16.64	214.76	−0.50	357.85	−13.72	225.63	4.54
RCA 3.5			317.45	−23.46	210.94	−2.27	321.47	−22.49	216.37	0.25
YCA 3.5			337.48	−18.63	208.68	−3.31	345.77	−16.63	219.06	1.50

**Table 10:** Breaking extension of undyed, water-dyed and SAE octane-dyed cotton samples.

Breaking Extension	Undyed				Water				SAE			
	Warp		Weft		Warp		Weft		Warp		Weft	
Sample	(mm)	(%)	(mm)	(%)	(mm)	(%)	(mm)	(%)	(mm)	(%)	(mm)	(%)
BCA 3.5	16.65	22.20	9.42	12.56	16.60	21.96	8.72	11.64	16.68	22.24	8.85	11.80
RCA 3.5					16.56	21.93	8.45	11.36	16.61	22.15	8.61	11.48
YCA 3.5					16.04	21.25	8.89	11.97	16.18	21.57	9.16	12.21

## Data Availability

All data generated and analysed during this study are included in this published article.
